# Chronic Pelvic Osteomyelitis: Case Report of a Rare Complication of Bone Marrow Biopsy

**DOI:** 10.7759/cureus.20599

**Published:** 2021-12-22

**Authors:** Vivek Bhat, Seetharam Anandram, Aaron C Lobo, Ashika Davis, Deepa S John

**Affiliations:** 1 Internal Medicine & Hematology, St. John's Medical College, Bangalore, IND; 2 Radiology, St. John's Medical College, Bangalore, IND

**Keywords:** iatrogenic complication, trephine biopsy, non-hodgkins lymphoma, chemotherapy, bone infection

## Abstract

Osteomyelitis commonly involves the long bones, with pelvic involvement uncommon. We report the case of a 50-year-old male who, following a bone marrow biopsy that diagnosed him with non-Hodgkin’s lymphoma, had persistent complaints of fever, swelling, and pain over the biopsy site. Pus cultures revealed growth of methicillin-resistant *Staphylococcus aureus* (MRSA), with computed tomography and magnetic resonance imaging of the pelvis revealing features of osteomyelitis of the right ilium. He was managed conservatively with antibiotics. On the last follow-up, he had just recovered from another flare of the infection. Bone marrow biopsy is a common tool in the hematologist's inventory. It is quite safe, with complications reported in less than 0.1% of all cases. Osteomyelitis of the pelvis following this is exceedingly rare; to our knowledge, only two prior such cases have been reported. Pelvic osteomyelitis is characterized by poorly defined hip pain, limited range of motion, and difficulty with ambulation. In case of intractable hip or buttock pain following a bone marrow biopsy, osteomyelitis of the pelvis must be considered in the differential diagnosis, and appropriate management must be begun. A multidisciplinary approach is required, with surgical debridement and appropriate antibiotics.

## Introduction

Osteomyelitis is an infection characterized by progressive inflammation, local destruction, and the apposition of new bone. It occurs due to hematogenous seeding, contiguous spread from a source of infection, or vascular insufficiency [1]. *Staphylococcus aureus* is the most commonly identified organism in these infections. Osteomyelitis of the pelvis is uncommon, constituting 2-11% of all osteomyelitis cases [2].

The bone marrow biopsy is a widely performed investigation in hematological disorders and is generally considered safe, with studies reporting an incidence of complications between 0.05% and 0.1% [3,4]. Infection as a complication is exceedingly rare. We report a case of pelvic osteomyelitis as a complication of bone marrow biopsy.

## Case presentation

A 50-year-old male, with no other comorbidities presented with a one-month history of fever and generalized fatigue. On evaluation, he had pancytopenia. He tested negative for human immunodeficiency virus (HIV). He was started on empirical vancomycin, amikacin, and piperacillin-tazobactam for neutropenic sepsis.

A bone marrow biopsy was performed in the right posterior super iliac spine. Based on morphology and immunohistochemistry (CD5, CD3, CD8, and CD4 positivity), he was diagnosed with T-cell non-Hodgkin's lymphoma. He was started on a regimen of cyclophosphamide, doxorubicin, vincristine, and prednisolone (CHOP regimen).

Four days after the procedure, a soft tissue swelling was noted at the biopsy site. Soft tissue ultrasonography showed features of hematoma, and this was managed conservatively.

Following his discharge, he continued to experience pain and swelling at the bone marrow site. Two weeks later, was readmitted with fever. Blood cultures did not show any growth, so he was initially started on empirical intravenous meropenem. Despite this, he continued to have pain and fever spikes, with pus discharge from the bone marrow site.

**Figure 1:**
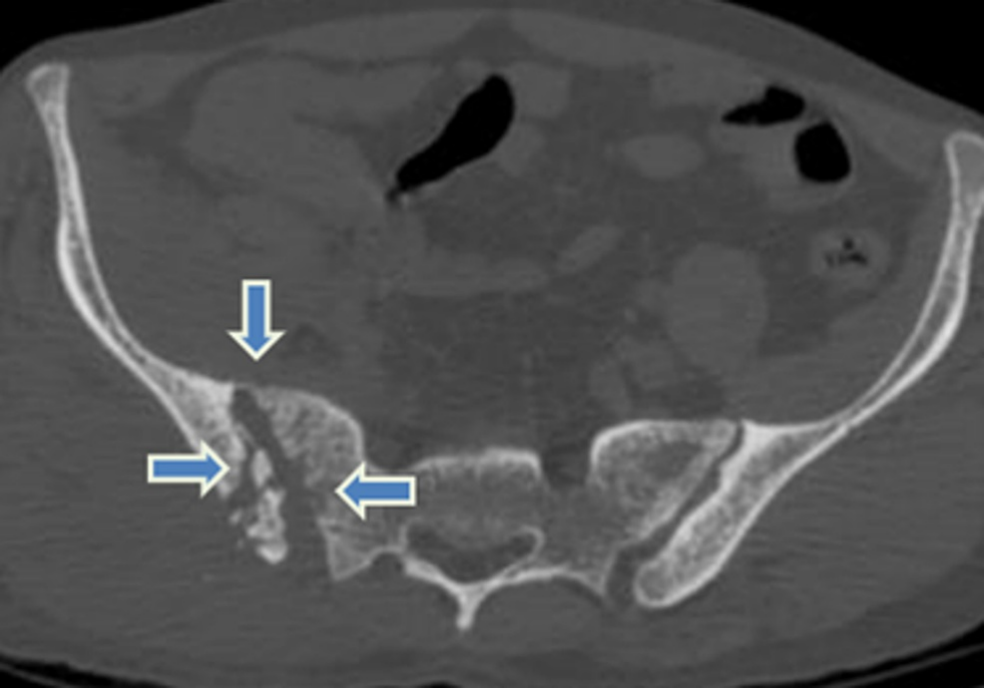
Axial CT of the pelvis in bone window, showing widening of the right sacroiliac joint (downward blue arrow) with focal erosions in the right sacral ala (leftward blue arrow) and sclerosis of the right iliac articular surface. Cloaca formation (rightward blue arrow) is noted in the iliac articular surface with sequestrum within the medullary cavity. CT - computed tomography

**Figure 2:**
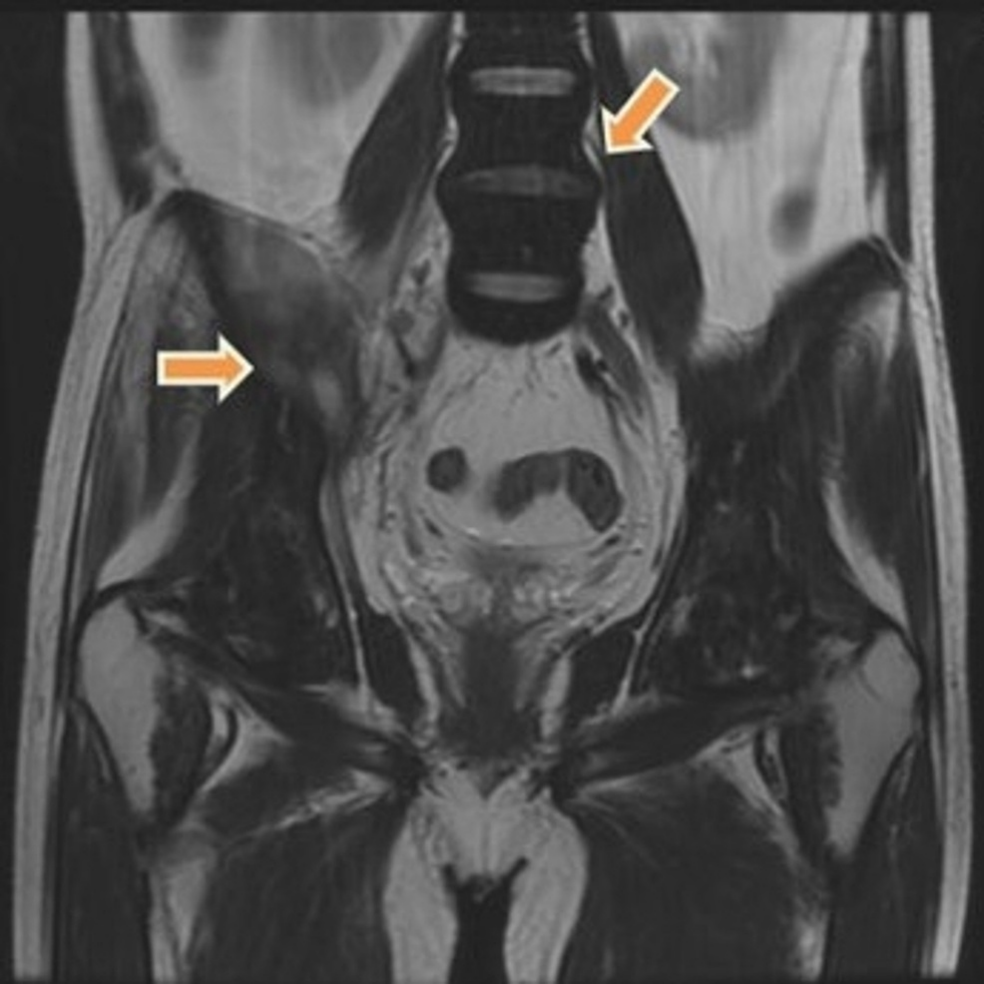
Coronal T2 weighted MRI image showing myositis of right iliopsoas muscles (rightward orange arrow) with diffuse marrow hypointensity (downward orange arrow) secondary to lymphomatous infiltration. MRI - Magnetic Resonance Imaging

Computed tomography and magnetic resonance imaging of the pelvis were ordered. These showed features of chronic osteomyelitis of the right iliac bone: erosions, cloaca, and sequestrum formation (Figures 1-2). Pus cultures revealed growth of methicillin-resistant *Staphylococcus aureus* (MRSA), sensitive to vancomycin.

Intravenous vancomycin was added and given for six weeks. He was discharged after becoming afebrile. Surgical intervention was deferred in view of pancytopenia and frailty. Meanwhile, he went into complete remission following the completion of six cycles of the CHOP regimen.

Over the next three years, he had a persistent low-grade fever, and he was admitted on multiple occasions for the treatment of his osteomyelitis, due to fever spikes and/ or pain over the biopsy site. Subsequent cultures grew *Pseudomonas* *aeruginosa, *sensitive to beta-lactams, so he was treated with intravenous piperacillin-tazobactam for six weeks.

**Figure 3:**
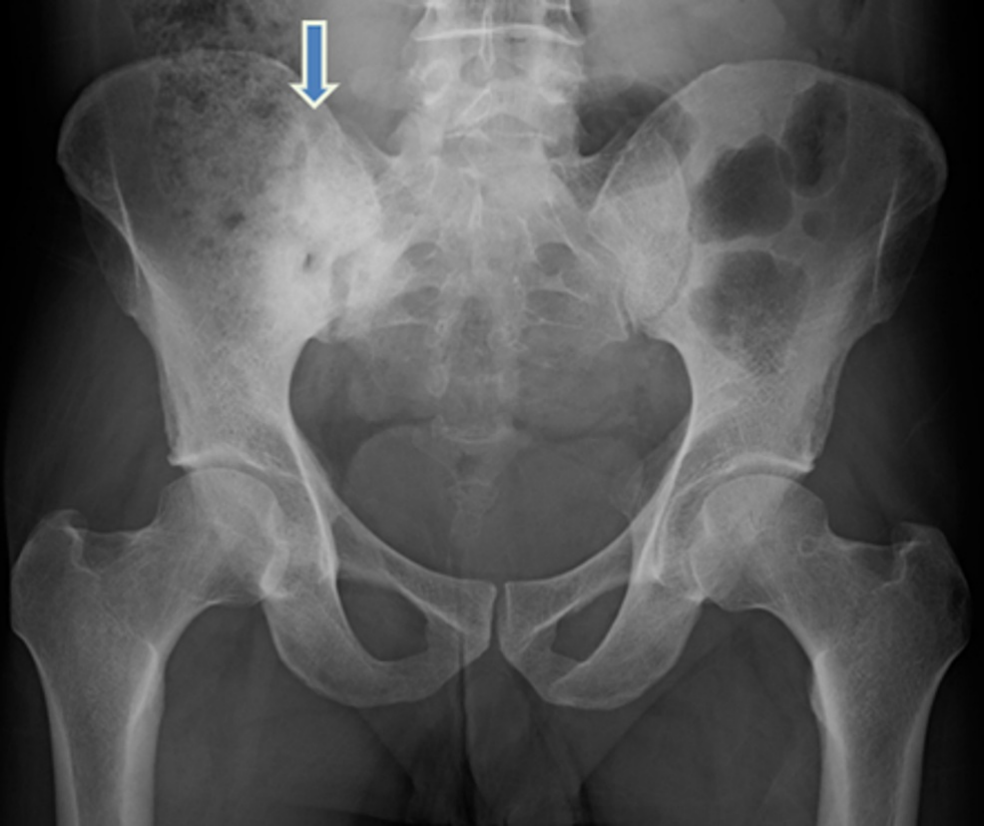
AP X-ray of the pelvis showing sclerosis of the sacroiliac joint margins with widening of the sacroiliac joint space (downward blue arrow). AP - Anteroposterior

**Figure 4:**
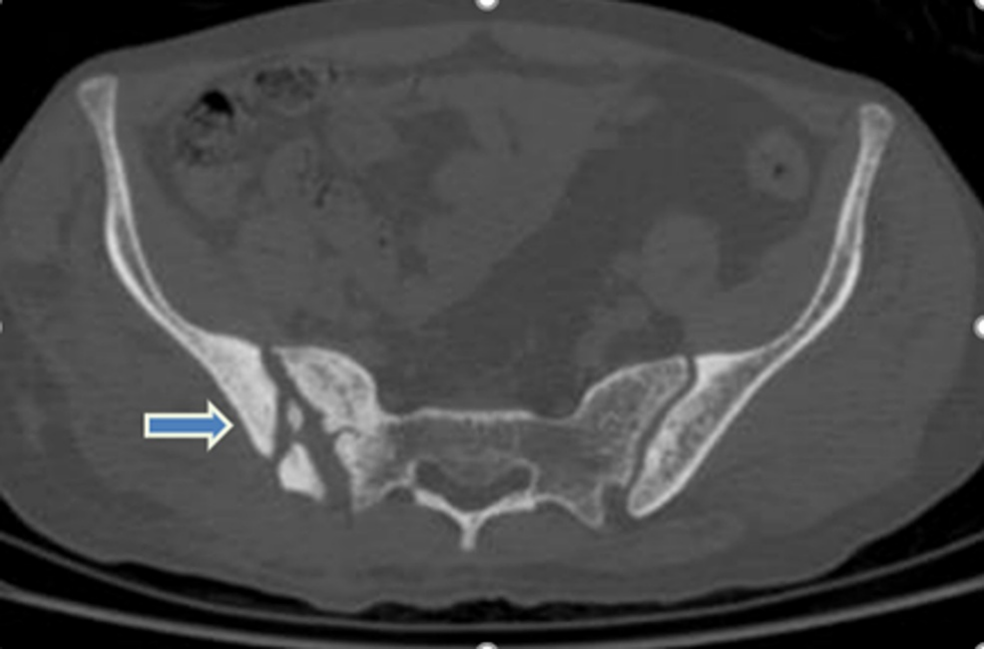
Axial CT of the pelvis after two years showing extensive sclerosis of the sacroiliac joint margins with widening of the sacroiliac joint space (rightward blue arrow). CT - Computed Tomography

Repeat imaging, two years later, continued to reveal features of active infection: sinus at the right gluteal region skin surface, an infective collection in the subcutaneous plane communicating via a tract with the right iliac bone, and extensive sclerosis of sacral and iliac articular surfaces (Figures 3-4). His lymphoma remained in remission.

On multiple occasions, he refused surgical intervention. On his last follow-up, he had just recovered from another flare of infection from the biopsy site, with pus culture growing methicillin-sensitive *Staphylococcus aureus.* He was treated with intravenous piperacillin-tazobactam for six weeks. 

## Discussion

The bone marrow biopsy - either trephine or aspiration - is a common investigation in the hematologist’s inventory. It is considered safe, with complications uncommon. Bain et al., in their surveys, found a total number of 43 complications from almost 35000 procedures. Bleeding, by far, was the most common complication. This was seen in our patient as well, who initially suffered a minor hematoma. However, Bain et al. only noted seven infections, none of which led to osteomyelitis, further highlighting our case's rarity [3,4]. 

Osteomyelitis commonly affects the long bones but can also affect short or flat bones such as those in the pelvis. Most cases in adults are localized to a single bone, usually due to a local risk factor - urologic surgery leading to osteomyelitis of the pubis, for example. Among the pelvic bones, the most commonly affected is the ilium [5]. Pelvic osteomyelitis is often characterized by vague symptoms of poorly defined hip pain, limited range of motion, and difficulty with ambulation [6], and often poses a diagnostic challenge, as the pain may be poorly localized due to the deep location of the bones involved [7]. Our patient presented with pain and swelling over the biopsy site, suggestive of iliac bone osteomyelitis. Infection is commonly polymicrobial. *Staphylococcus aureus* is the most commonly observed organism, as also seen in our case [2].

Studies have described pelvic osteomyelitis complicating pelvic surgery [8], or chronic decubitus ulcers [9], among other causes. To our knowledge, this is the third case of pelvic osteomyelitis due to a bone marrow biopsy reported in English literature [10,11], and the fourth case of osteomyelitis complicating a bone marrow biopsy [12]. Khakwani et al. described the case of a 51-year-old male, being treated for chronic lymphocytic leukemia, who presented with increasing right buttock pain and fever, six weeks after undergoing a bone marrow biopsy. MRI revealed acute osteomyelitis of the right ilium and sacrum, along with septic arthritis of the right sacroiliac joint [10]. Tural-Kara et al. described the case of an 18-month-old boy with familial Mediterranean fever, who presented with complaints of left pelvic pain, and difficulty walking without support, three months after a bone marrow biopsy. Imaging revealed left-sided sacroiliac osteomyelitis, and an iliopsoas abscess [11]. Finally, Shah et al. reported sternal osteomyelitis after a marrow biopsy from the sternum [12]. All three prior cases isolated *Staphylococcus aureus*, but methicillin resistance was absent, unlike in our case. All cases were treated successfully with appropriate intravenous and oral antibiotics [10-12].

The treatment of pelvic osteomyelitis can be complex due to the anatomical constraints of the pelvis, and the high degree of comorbidity in these patients [13]. A multidisciplinary approach is often required [5,13], with surgical excision of all necrotic tissue in addition to broad-spectrum antibiotics covering *Staphylococcus aureus*, coliforms, and gram-negative rods for a variable duration [5]. In our patient, we observed monomicrobial infections, so we administered appropriate antibiotics guided by culture and sensitivity. In many cases, without surgical intervention, complete resolution is unlikely, as seen in our patient. Complicated cases may require hemipelvectomy or hemicorporectomy, procedures that carry significant mortality [6].

## Conclusions

Due to its rarity, pelvic osteomyelitis is rarely considered in cases of intractable pain over the hip or buttock. While the bone marrow biopsy is a safe procedure, it may cause unexpected complications, including infections. Therefore, in a patient with suspicious complaints following a bone marrow biopsy, osteomyelitis must be considered. Appropriate investigations must be ordered to confirm the diagnosis, and appropriate antibiotics must be given in order to limit the significant morbidity that can result.
